# Complete Genome Sequence of *Marinobacter* sp. Strain JH2, Isolated from Seawater of the Kiel Fjord

**DOI:** 10.1128/MRA.00596-19

**Published:** 2019-07-25

**Authors:** Jacqueline Hollensteiner, Anja Poehlein, Rolf Daniel

**Affiliations:** a Genomic and Applied Microbiology and Göttingen Genomics Laboratory, Institute of Microbiology and Genetics, University of Göttingen, Göttingen, Germany; Indiana University, Bloomington

## Abstract

Here, we present the genome sequence of the Gram-negative and rod-shaped *Marinobacter* sp. strain JH2, which was isolated from seawater of the Kiel Fjord in Germany. The draft genome consists of two replicons, including one chromosome (3.6 Mb) and a circular plasmid (36.7 kb). The genome harbors 3,347 protein-coding genes.

## ANNOUNCEMENT

*Marinobacter* sp. strain JH2 is a Gram-negative, moderately halophilic, and rod-shaped bacterium. It was isolated from 200 μl seawater of the Kiel Fjord (Germany). Members of the genus *Marinobacter* are well distributed in aquatic environments all over the world (1–4). One feature of *Marinobacter* spp. is their ability to utilize various hydrocarbons (5–7). The genome sequence of *Marinobacter* sp. JH2 provided insights into the biocatalytic potential related to hydrocarbon degradation.

*Marinobacter* sp. JH2 was isolated and grown in Difco marine broth 2216 (BD Biosciences, Heidelberg, Germany) at 28°C. Genomic DNA was extracted using the MasterPure complete DNA purification kit, as recommended by the manufacturer (Epicentre, Illumina, Madison, WI, USA). The isolated DNA was used to generate Illumina shotgun paired-end sequencing libraries. The MiSeq system and the MiSeq reagent kit v3 (600 cycles) were used for sequencing, as recommended by the manufacturer (Illumina, San Diego, CA, USA). Additionally, the 1D genomic DNA sequencing protocol for the MinION device using SQK-LSK109 was conducted (Oxford Nanopore, Oxford, UK). End repair was performed using NEBNext formalin-fixed, paraffin-embedded (FFPE) repair mix (New England Biolabs, Ipswich, MA, USA). The library for Nanopore sequencing was loaded on a SpotON flow cell Mk I (R9.4). Reads were quality filtered using fastp v0.19.4 (8), which resulted in 6,291 long reads (Nanopore) and 2,704,110 short reads (Illumina). The bacterial genome assembler Unicycler v0.4.8-beta (9) was used to perform a *de novo* hybrid genome assembly, which resulted in a 135-fold coverage. The assembly was validated with Bandage v0.8.1 (10). The complete genome sequence consists of one circular chromosome (3,686,730 bp) and an overall G+C content of 53.55%. Additionally, one circular plasmid (36,788 bp) with a G+C content of 50.44% was identified. The genome annotation was performed with the Rapid Prokaryotic Genome Annotation (Prokka) tool v1.13.3 (11). The predicted 3,413 genes included 53 tRNA genes, 12 rRNA genes, 1 transfer-messenger RNA (tmRNA) gene, and 3,347 protein-encoding genes. The relationship to other members of the genus *Marinobacter* was determined by calculating the average nucleotide identity (ANI) with the Python module for average nucleotide identity analyses (pyANI) v0.2.7 (12). The 35 genome sequences of all species belonging to the genus *Marinobacter* available at National Center for Biotechnology Information (accessed 12 March 2019) were included in the analysis. The analysis revealed that *Marinobacter*. sp. JH2 was most closely related to Marinobacter vinifirmus strain Z-F7-3 and Marinobacter litoralis strain Sw-45, with an ANI value of 87.6%. This value indicates that *Marinobacter* sp. JH2 represents a new species group within the genus *Marinobacter*.

Genome analysis revealed the presence of the complete *alk* gene cluster (*alkSB1GHJ*) for hydrocarbon catabolism (13). Similarity analysis with the *alk* gene of the hydrocarbonoclastic Alcanivorax borkumensis SK2 revealed that the genome of *Marinobacter* sp. JH2 harbors the genes encoding AlkS, AlkB1, AlkG, AlkH, and AlkJ (see locus tags MARI_17890 to MARI_17930), and the recorded amino acid sequence identities were 73.83%, 87.16%, 68.97%, 84.92%, and 89.49%, respectively.

### Data availability.

The whole-genome shotgun project of *Marinobacter* sp. strain JH2 has been deposited at DDBJ/EMBL/GenBank under the accession numbers CP037934 and CP037935, with BioProject accession number PRJNA526490 and SRA accession number SRP199986.
